# Epigenetic repression of ROR2 has a Wnt-mediated, pro-tumourigenic role in colon cancer

**DOI:** 10.1186/1476-4598-9-170

**Published:** 2010-06-30

**Authors:** Ester Lara, Vincenzo Calvanese, Covadonga Huidobro, Agustin F Fernández, Ángela Moncada-Pazos, Álvaro J Obaya, Oscar Aguilera, José Manuel González-Sancho, Laura Sánchez, Aurora Astudillo, Alberto Muñoz, Carlos López-Otín, Manel Esteller, Mario F Fraga

**Affiliations:** 1 Department of Immunology and Oncology, National Center for Biotechnology, CNB-CSIC, Cantoblanco, Madrid E-28049, Spain; 2 Cancer Epigenetics Laboratory, Instituto Universitario de Oncología del Principado de Asturias (IUOPA), HUCA, Universidad de Oviedo, 33006-Oviedo, Spain; 3 Departamento de Bioquímica y Biología Molecular, Instituto Universitario de Oncología, Universidad de Oviedo, 33006-Oviedo, Spain; 4 Instituto de Investigaciones Biomédica Alberto Sols, Consejo Superior de Investigaciones Científicas, Universidad Autónoma de Madrid, E-28029 Madrid, Spain; 5 Banco de Tumores. Instituto Universitario de Oncología del Principado de Asturias (IUOPA), HUCA, Universidad de Oviedo, 33006-Oviedo, Spain; 6 Cancer Epigenetics and Biology Program (PEBC), Bellvitge Biomedical Research Institute (IDIBELL), Barcelona, Spain

## Abstract

**Background:**

Wnt factors control cell differentiation through semi-independent molecular cascades known as the β-catenin-dependent (canonical) and -independent (non-canonical) Wnt signalling pathways. Genetic and epigenetic alteration of components of the canonical Wnt signalling pathway is one of the primary mechanisms underlying colon cancer. Despite increasing evidence of the role of the non-canonical pathways in tumourigenesis, however, the underlying molecular mechanisms are poorly understood.

**Results:**

Here we report that the receptor tyrosine kinase-like orphan receptor 2 (ROR2), a transmembrane receptor for Wnt factors that activates non-canonical pathways, is frequently repressed by aberrant promoter hypermethylation in human colon cancer cell lines and primary tumours. By restoring ROR2 activity in colon cancer cells harbouring *ROR2 *promoter hypermethylation, we show that the role of ROR2 in colon cancer cells is mediated, at least in part, by canonical Wnt and that its epigenetic-dependent loss can be pro-tumourigenic.

**Conclusions:**

Our data show the importance of epigenetic alterations of ROR2 in colon cancer, highlighting the close interconnection between canonical and non-canonical Wnt signalling pathways in this type of tumour.

## Introduction

The receptor tyrosine kinase-like orphan receptor 2 (ROR2) is a transmembrane protein that belongs to a conserved family of tyrosine kinase receptors involved in many developmental processes, including chondrogenesis [1], osteoblastogenesis [2] and neural differentiation [3]. Accordingly, *ROR2 *mutations in humans result in dominant brachydactyly type B and Robinow syndrome [4], two syndromes of altered development characterised by short stature, brachydactyly, segmental defects of the spine and dysmorphic facial appearance [5].

ROR2 exerts its role in cell differentiation primarily through the Wnt signalling pathway [6]. This pathway is comprised of a number of extracellular effectors, membrane proteins, intracellular signal transducers and nuclear gene regulators that transmit extracellular signals to the nucleus as precise instructions for regulating specific genes [7]. When β-catenin participates in this cascade, the signalling pathway is known as canonical Wnt. Wnt effectors can also induce β-catenin-independent signals that make up the non-canonical Wnt signalling pathway. Within the Wnt signalling pathway, the primary role of ROR2 is to mediate WNT5A signals in a complex manner that is still unclear. ROR2 was initially shown to mediate WNT5A-dependent inhibition of canonical Wnt signalling downstream of β-catenin stabilisation in 293 cells, at the level of TCF-mediated transcription [8]. ROR2 was subsequently shown to mediate WNT5A-dependent JNK activation in regulating convergent extension movements in *Xenopus *gastrulation [9], and is also known to enhance WNT1 and antagonise WNT3 activities in osteoblastic cells [10]. In the H441 lung carcinoma cell line, ROR2 positively modulates Wnt3a-activated canonical signalling [11].

The Wnt signalling pathway is central to cell differentiation and cancer. Genetic and epigenetic alterations of components of the canonical Wnt signalling pathway are a primary mechanism of colon cancer development [7]. ROR2 is overexpressed in oral [12] and renal cancer [13], and in osteosarcoma [14]. ROR2 overexpression activates JNK, a component of the non-canonical Wnt pathway, and has pro-tumourigenic effects [13,14].

ROR2 also mediates inhibition of the β-catenin-dependent Wnt signalling pathway [8,10,15]. Paradoxically, the aberrant epigenetic repression of other Wnt inhibitors such as WIF-1, DKK1, SFRP1 and SFRP2 directly promotes tumourigenesis in colon cancer cells by promoting constitutive Wnt signalling [7,16-18]. Indeed, the ROR2 extracellular ligand WNT5A, which inhibits the canonical Wnt signalling pathway in certain molecular contexts [8], is also aberrantly repressed by promoter hypermethylation in acute lymphoblastic leukaemia [19] and in colon cancer [20], and its absence is tumourigenic in these tumour types. As ROR2 mediates the inhibition of canonical signalling by WNT5, we hypothesised that this orphan receptor could also be a target of aberrant epigenetic regulation in colon cancer. Here we report that ROR2 is frequently repressed by promoter hypermethylation in colon cancer and that its loss can be protumourigenic in colon cancer.

## Results

### ROR2 promoter is frequently aberrantly hypermethylated in colon cancer

**Figure 1:**
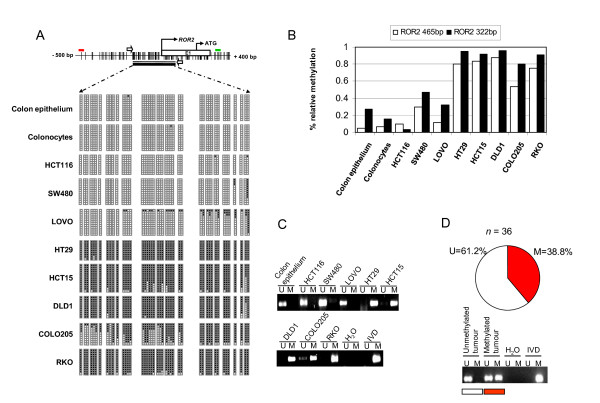
**Aberrant *ROR2 *hypermethylation in colon cancer**. (A) The *ROR2 *promoter with its associated CpG island (top). CpG location, transcription start site (TSS) and ATG are indicated. Bisulphite sequencing primers, white arrows; Illumina Infinium probes (465 bp upstream and 322 bp downstream of the TSS), red and green lines, respectively. Lower panels show bisulphite genomic sequencing results of 12 individual clones in healthy colon epithelium, colonocytes and eight colon cancer cell lines (HCT116, SW480, LOVO, HT29, HCT15, DLD1, COLO205 and RKO), showing methylated (black squares) or unmethylated cytosines (white squares). (B) DNA methylation analysis of two CpG sites within the *ROR2 *promoter in healthy colon epithelium, colonocytes and eight colon cancer cell lines using Infinium methylation arrays. Relative methylation for each CpG site (-465 bp and + 322 bp) is represented on a scale from 0.0 to 1.0 (corresponding to a 0% and 100% likelihood of CpG hypermethylation). (C) Methylation-specific PCR (MSP) of ROR2 promoter in healthy colon tissue and colon cancer cell lines. A PCR band in lanes M or U indicates methylated or unmethylated genes, respectively. *In vitro*-methylated DNA (IVD) was used as a positive control for methylated DNA. (D) Methylation analysis of the ROR2 promoter using MSP of 36 primary colon tumours. Percentage of tumours with ROR2 promoter hypermethylation (unmethylated, white; methylated, red; top) and ethidium bromide staining of an MSP reaction of two representative tumours (unmethylated and methylated) resolved in an agarose gel (bottom).

Analysis of the region 1.0 kb upstream and 0.5 kb downstream of the ROR2 transcriptional start site identified a CpG island, suggesting a potential role for CpG methylation in the regulation of ROR2 expression. To study the possible aberrant epigenetic regulation of ROR2 in colon cancer, we used bisulphite sequencing of multiple clones to determine the methylation status of a *ROR2 *promoter DNA region of 315 bp that spans the ROR2 transcriptional start point in healthy colon tissue, *in vitro*-growing colonocytes and eight colon cancer cell lines (HCT116, SW480, LOVO, HT29, HCT15, DLD1, COLO205 and RKO) (Figure 1A). This showed that the *ROR2 *promoter was completely unmethylated in non-tumourigenic colon primary tissues and *in vitro*-growing colonocytes, whilst it was densely hypermethylated in most cancer cell lines analysed (HT29, HCT15, DLD1, COLO205 and RKO). These results were confirmed using the 27K Illumina Infinium methylation platform to study the DNA methylation status of two CpG positions (located 465 bp upstream and 322 bp downstream of the ROR2 transcription start site, respectively) within the ROR2 CpG island (Figure 1B) and using methylation-specific PCR (Figure 1C).

To determine whether *ROR2 *promoter hypermethylation is also a frequent *in vivo *event, we used methylation-specific PCR to analyse 36 primary colon adenocarcinomas (Figure 1D). We detected methylation at the *ROR2 *promoter in 14 of the 36 tumours (38.8%), which confirmed that this is a common event *in vivo*.

### Promoter DNA methylation-mediated ROR2 repression in colon cancer

**Figure 2:**
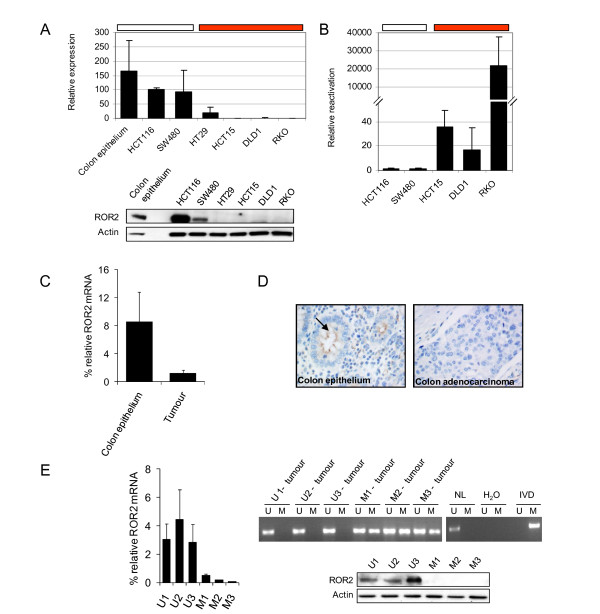
**Promoter methylation-dependent ROR2 repression in colon cancer**. (A) qRT-PCR analysis of ROR2 mRNA relative to GAPDH in three samples with unmethylated ROR2 (white) and four samples with ROR2 promoter hypermethylation (red; top panel). The bottom panel shows WB analysis of ROR2 and actin in normal colon epithelium. Two samples show unmethylated ROR2 (HCT116, SW480) and four samples show ROR2 promoter hypermethylation (HT29, HCT15, DLD1, RKO). (B) Relative ROR2 mRNA levels in two cancer cell lines with unmethylated ROR2 (white) and three samples with ROR2 promoter hypermethylation (red) after treatment with the demethylating drug 5-aza-2'-deoxycytidine. (C) qRT-PCR analysis of ROR2 mRNA in 20 primary colorectal tumours and the corresponding normal colon epithelium. Results are shown as the mean ± SD of three independent experiments. (D) Immunohistochemistry analysis of ROR2 in formalin-fixed, paraffin-embedded tissues. The images show protein expression in colorectal gland mucosa (arrow) in normal colon epithelium and lack of ROR2 expression in a poorly differentiated colon adenocarcinoma. (E) Relationship between ROR2 expression and promoter hypermethylation. qRT-PCR quantification of ROR2 relative to GAPDH expression in three ROR2-expressing tumours (U1-3) and three tumours with low ROR2 expression (M1-3; left panel). MSP analysis of ROR2 promoter methylation. A PCR band in lanes M or U indicates methylated or unmethylated, respectively. IVD is used as a positive control for methylated DNA and normal lymphocytes (NL) as negative control (upper right). WB analysis of ROR2 protein expression in the same tumour samples (lower right).

To determine the role of the *ROR2 *promoter hypermethylation in gene expression, we used qRT-PCR and WB to compare ROR2 mRNA and protein levels in healthy colon epithelium and the HCT116 and SW480 cell lines, which do not show *ROR2 *promoter hypermethylation, with the HT29, HCT15, DLD1 and RKO lines, which present dense DNA methylation at the *ROR2 *promoter (Figure 2A). These experiments showed that ROR2 mRNA and protein were only detected in samples lacking *ROR2 *promoter hypermethylation. Although they showed dense *ROR2 *promoter hypermethylation, HT29 cells expressed residual levels of ROR2 mRNA, insufficient to detect ROR2 protein. To study the relationship between *ROR2 *promoter hypermethylation and ROR2 repression in more detail, we analysed ROR2 mRNA levels in colon cancer cell lines incubated with the demethylating drug 5-aza-2-deoxycytidine (Figure 2B). Treatment with this drug resulted in marked reactivation of ROR2 in cell lines displaying *ROR2 *promoter hypermethylation (HCT15, DLD1 and RKO), but not in those lacking hypermethylation (HCT116 and SW480) (Figure 2B). This implies that *in vitro ROR2 *promoter hypermethylation is directly associated with ROR2 repression.

To study the relationship between *ROR2 *promoter hypermethylation and gene expression *in vivo*, we analysed ROR2 mRNA levels and used tissue microarrays to determine the levels of ROR2 protein in 20 pairs of normal and tumour colon tissues obtained from the same patients (Figures 2C, D). At the mRNA level, in 18 of the 20 paired samples analysed, ROR2 was much less strongly expressed in tumour tissues than in their normal counterparts; mean ROR2 expression was seven times higher in colon epithelium than in colon tumours (Figure 2C). At the protein level, we also observed lower ROR2 levels in tumour samples in most cases; we only detected positive ROR2 staining in 35% of the colorectal tumours analysed (Figure 2D). To determine the relationship between *ROR2 *promoter hypermethylation with mRNA and protein expression, we selected three tumours that expressed and three that did not express ROR2 mRNA, and analysed the methylation status of the *ROR2 *promoter by methylation-specific PCR (Figure 2E) and ROR2 protein levels by WB. The results confirmed that tumours lacking ROR2 expression presented promoter hypermethylation and lack ROR2 protein. This corroborated a relationship between *ROR2 *promoter hypermethylation and gene repression *in vivo*. The data indicate that *ROR2 *promoter hypermethylation is directly associated with ROR2 repression in colon cancer, both *in vitro *and *in vivo*.

### Aberrant ROR2 promoter hypermethylation can promote tumourigenesis in colon cancer cells

**Figure 3:**
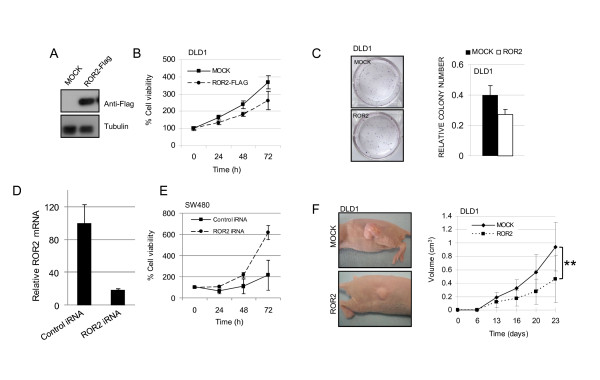
**Restoration of ROR2 expression impairs tumour growth *in vitro *and *in vivo***. The effect of ROR2 restoration was studied in the DLD1 colon cancer cell line. (A) WB showing restoration of ROR2 after transfection with the pcDNA3.1-ROR2-Flag construct; anti-Flag antibody was used. (B) MTT analysis of cell viability in DLD1 mock-(squares) and ROR2-Flag-transfected (circles) cells. (C) Colony formation assay of DLD1-transfected cell line. DLD1 mock-and ROR2-Flag-transfected cells were seeded in six-well plates and cultured for two weeks, when colonies were visible. Colony number is expressed relative to number of cells seeded. (D) Quantitative RT-PCR analysis of ROR2 mRNA relative to GAPDH in the SW480 cell line transfected with a control siRNA (control iRNA) or a ROR2-specific siRNA (ROR2 iRNA). (E) MTT analysis of viability in SW480 cells expressing ROR2 (control iRNA, squares) or ROR2 knocked-down (ROR2 iRNA, circles). (F) Effect of restoration of ROR2 expression on *in vivo *tumour growth. Mouse xenograft model, in which DLD1 mock-transfected cells were injected subcutaneously into the left flank and ROR2-transfected cells into the right flank of athymic nude-Foxn1_nu/un _mice; representative images are shown (left). The right panels shows quantification of tumour size (cm^3^) in the mouse xenograft model; DLD1 mock-(circles) and ROR2-transfected cells (squares). (** *p *= < 0.01).

To evaluate the functional role of ROR2 repression by promoter hypermethylation in colon cancer, we transfected ROR2 in the colon cancer cell line DLD1, which shows DNA methylation-dependent ROR2 inactivation (Figure 3A). MTT analysis of the effects of ROR2 restoration on cell proliferation showed that ROR2 restoration induced a notable reduction in DLD1 cell proliferation (Figure 3B). We used colony-formation assays to further characterise the role of ROR2 in the growth of colon cancer cells, and found that restoration of ROR2 activity resulted in a 30% reduction in colony formation in DLD1 cells (Figure 3C). We also knocked down ROR2 expression by RNA interference in a ROR2-expressing colon cancer cell line unmethylated at the ROR2 CpG island, SW480. Reduced ROR2 expression (Fig. 3D) was associated with increased cell growth (Fig. 3E).

To study the *in vivo *role of ROR2 in colon carcinogenesis, we subcutaneously injected DLD1 cells with restored ROR2 expression and isogenic controls transfected with empty vectors into a cohort of immunodeficient nude mice (Figure 3F). In both cases, macroscopic tumours were observed 13 days after injection (Figure 3F). Restoration of ROR2 expression in DLD1 cells resulted in a marked reduction of tumour formation *in vivo*; at the time the mice were sacrificed, DLD1 tumours expressing ROR2 had on average half the volume of those lacking ROR2 activity (Figure 3F). This *in vivo *evidence links *ROR2 *aberrant promoter hypermethylation with colon cancer development.

### Canonical Wnt mediates the functional role of aberrant ROR2 promoter hypermethylation in colon cancer cells

**Figure 4:**
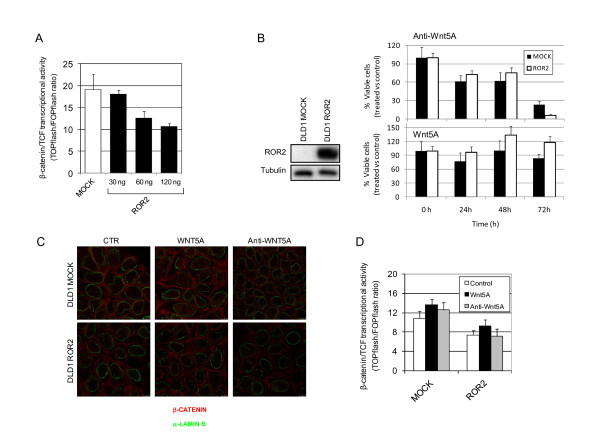
**Wnt-dependent role of ROR2 in colon cancer cells**. (A) Restoration of ROR2 inhibits β-catenin/TCF transcription activity. DLD1 colon cancer cells were transiently transfected with TOPflash or FOPflash reporters alone (white) or in the presence of different amounts of an ROR2 expression construct (black). Cells were lysed 48 h post-transfection and *Firefly *and *Renilla *luciferase activities measured. Results are shown as means ± SD of triplicate samples from one representative experiment. (B) WB showing ROR2 expression in DLD1 mock-and ROR2-transfected cells (left). MTT analysis of viability of the same cells in the presence of anti-WNT5A antibody (2 μg/ml; upper right) or recombinant WNT5A (200 ng/ml; lower right). Treatment was renewed daily. Data are expressed as the percent MTT signal relative to the untreated sample at the same time point. (C) Immunofluorescence staining of β-catenin and α-lamin B in DLD1 mock-and ROR2-transfected cells after 2 h treatment with recombinant WNT5A (200 ng/ml) or anti-WNT5A antibody (2 μg/ml) or untreated (CTR). (D) TOPflash assay (as in A) of the same cells after 72 h treatment (as in B) with recombinant WNT5A or anti-WNT5A antibody.

Epigenetic repression of other Wnt pathway components promotes tumourigenesis by the constitutive activation of canonical Wnt [7]. In addition, ROR2 regulates TCF-mediated transcription [8]. We thus hypothesised that the role of ROR2 in colon cancer is mediated by canonical Wnt signalling. To test this possibility, we tested whether its restoration interfered with β-catenin/TCF-dependent transcription, using luciferase reporter assays to analyse the effect of exogenous ROR2 expression in ROR2-silenced DLD-1 cells (Figure 4A). Restoration of ROR2 expression reduced basal β-catenin/TCF-dependent transcription in DLD1 cells by 50%, suggesting that the role of aberrant epigenetic repression of ROR2 in colon cancer is mediated, at least in part, by canonical Wnt signalling.

ROR2 is reported to mediate the WNT5A-dependent inhibition of β-catenin-TCF-regulated genes in human embryonic cells [8,15]. To determine whether the canonical Wnt-dependent role of aberrant epigenetic repression of ROR2 in colon cancer is WNT5A-associated, we used we used a dual approach: i) we increased extracellular levels using recombinant WNT5A protein and ii) we used anti-WNT5A antibody to greatly reduce WNT5A activity in DLD1 cells expressing ROR2 and control cells. We studied the effect of WNT5A modulation on cell growth and the Wnt signalling pathway. MTT assays showed that, independently of ROR2 expression, WNT5A removal reduced cell growth (Figure 4B, top). The decrease in cell growth was greater in DLD1 cells that did not express ROR2, although the difference was not significant (p = 0.12). Addition of recombinant WNT5A to culture medium had no relevant effect on cell growth, independently of ROR2 expression (Figure 4B, bottom).

To study the effect of WNT5A modulation on canonical Wnt signalling, we treated ROR2-expressing DLD1 cells or control DLD1 cells with anti-WNT5A antibody or with recombinant WNT5A. We determined nuclear β-catenin levels in immunofluorescence experiments (Figure 4C) and analysed β-catenin/TCF-dependent transcription in luciferase reporter assays (Figure 4D). Increasing or decreasing extracellular WNT5A levels had little impact on β-catenin nuclear location and β-catenin/TCF-dependent transcription (Figure 4C and 4D), suggesting that the canonical Wnt-dependent role of ROR2 epigenetic deregulation in DLD1 colon cancer cells does not necessarily require WNT5A.

## Discussion

We report that the receptor tyrosine kinase-like orphan receptor 2 (ROR2), a transmembrane protein that participates in Wnt signalling, is frequently repressed by aberrant promoter hypermethylation in human colon cancer. In addition, we demonstrate that the epigenetic-dependent loss of ROR2 can promote tumour growth in colon cancer cells. This effect can be mediated, at least in part, by the canonical Wnt signalling pathway, since restoration of ROR2 activity in DLD1 colon cancer cells induced a decrease in β-catenin/TCF-dependent transcription.

ROR2 is reported to have oncogenic properties in other tumour types such as oral cancer [12], renal cancer [13] and osteosarcoma [14]; it is frequently overexpressed in these tumours, and its suppression in renal cancer cells inhibits cell migration and growth in orthotopic xenograft models [13]. In osteosarcoma cells, suppressed expression of ROR2 (or its extracellular effector, WNT5A) inhibits cell invasiveness and decreases invadopodium formation [14].

These lines of evidence suggest that the role of ROR2 in cancer is complex and that it can either promote or suppress tumour formation, depending on tumour type and molecular context. A possible explanation for this complexity could be the multifaceted role of ROR2 in the Wnt signalling pathway. On the one hand, ROR2 can mediate WNT5A-dependent activation of JNK, a member of the non-canonical Wnt pathway in mice [9,21], while on the other, ROR2 can govern the WNT5A-dependent inhibition of canonical Wnt signalling downstream of β-catenin stabilisation [8]. Depending on the tumour type, ROR2 signals can therefore show a preference for β-catenin/TCF-dependent genes or for non-canonical Wnt pathways. Two lines of evidence support these possibilities: first, the pro-tumourigenic role of ROR2 in renal cancer and osteosarcoma is thought to be mediated by activation of the non-canonical Wnt signalling kinase JNK [13,14], and second, in colon cancer cells with constitutive Wnt signalling activity, restoration of ROR2 activity increased the inhibition of β-catenin reporter genes (our data and [15]).

As ROR2 is reported to mediate the WNT5A-dependent inhibition of β-catenin-TCF-regulated genes in human embryonic cells [8,15], we tested whether WNT5A mediated the canonical Wnt-dependent role of aberrant epigenetic ROR2 repression in colon cancer. We evaluated the effect of increased or decreased extracellular WNT5A levels on cell growth and canonical Wnt pathway status in ROR2-expressing DLD1 cells and controls. Although modulation of extracellular WNT5A levels affected cell growth, this effect was little influenced by ROR2 expression. The canonical Wnt pathway did not appear to be the effector of WNT5A modulation in these cells. Our data thus suggest that the canonical Wnt-dependent role of ROR2 hypermethylation in colon cancer cells does not necessarily require WNT5A. As WNT5A might have canonical and non-canonical effects, WNT5A/ROR2 could have a role in colon cancer through non-canonical Wnt signalling within certain molecular contexts. This pathway is frequently altered in other tumour types [12-14]. Our results nonetheless do not rule out a role for WNT5A in colon cancer through the canonical Wnt signalling pathway; indeed, WNT5A inhibits β-catenin/TCF-dependent transcription in HCT116 colon cancer cells [20].

As proposed for ROR2, WNT5A might also have either a tumour-promoting or-suppressing role. Many studies report a tumour-suppressing effect, and it is downregulated in a number of cancers such as colorectal and ductal breast cancer, neuroblastoma and leukaemia (reviewed in [22]). As we show here for ROR2, WNT5A repression in colon and haematopoietic tumours is mediated by aberrant promoter hypermethylation [19,20]. In contrast, WNT5A also has a tumour-promoting role in cases such as non-small-cell lung cancer, melanoma, breast, gastric, pancreatic and prostate cancers (reviewed in [22]). As ROR2 and WNT5A functions are intimately associated--indeed, ROR2 knockout mice phenocopy most of alterations seen in WNT5A knockout mice [23]--ROR2 and WNT5A might also have parallel or complementary roles in cancer. ROR2/WNT5A upregulation could be advantageous to cancers driven by non-canonical Wnt signalling, while their epigenetic downregulation would benefit tumours, such as colon and haematopoietic cancers, that are driven by canonical Wnt signalling.

## Methods

### Human cancer cell lines culture and treatments, and primary tumour samples

The eight human colon cancer cell lines (HCT116, SW480, LOVO, HT29, HCT15, DLD1, COLO205 and RKO) and healthy colonocytes were obtained from the American Type Culture Collection. Cell lines were maintained in appropriate medium and treated with 2 μmol/L 5-aza-2'-deoxycytidine (Sigma) for 3 days for DNA demethylation, anti-WNT5A antibody (2 μg/ml; R&D) or recombinant WNT5A (200 ng/ml; Millipore). We obtained 36 primary colon tumours from the Spanish National Cancer Research Centre Tumour Bank and 20 pairs of same-patient healthy and colon tumour tissue from the Institute of Oncology of Asturias Tumour Bank. The study was approved by the appropriate institutional review boards.

### DNA methylation analysis of the ROR2 gene

We established ROR2 CpG island methylation status by PCR analysis of bisulphite-modified genomic DNA. Methylation status was first analysed by bisulphite genomic sequencing of the CpG island using primers: 5'-GGG GTT TTA GTT GTA GTT TTA GT-3' (sense) and 5'-CTC CTC CTT CTC CCT AAC-3' (anti-sense). Twelve independent clones were sequenced for each sample. The second analysis used methylation-specific PCR with primers specific to the methylated or modified unmethylated DNA. Primer sequences for the methylated reaction were 5'- GTT TCG TTT TGT TTA TCG GGG C - 3' (sense) and 5'-ACT AAA AAA ATT CCT TAA CGC GAA -3' (anti-sense), and for the unmethylated reaction 5'-GTT TTG TTT TGT TTA TTG GGG TGG-3' (sense) and 5'-TAA CTA AAA AAA TTC CTT AAC ACA AA-3' (anti-sense). *In vitro*-methylated DNA (IVD) was used as a positive control for the methylated reaction. PCR products were identified in ethidium bromide-stained 2% agarose electrophoresis gels and viewed under UV light. We designed the bisulphite genomic sequencing and methylation-specific PCR primers using Methyl Primers Express software and confirmed bisulphite sequencing and methylation-specific PCR results using Illumina Infinium Methylation Arrays (Illumina). The two CpG sites analysed were located 465 bp upstream and 322 bp downstream of the *ROR2 *transcriptional start point. Relative DNA methylation for each CpG site is represented by a scale from 0.0 to 1.0 (corresponding to 0% and 100% likelihood of gene promoter hypermethylation, respectively).

### ROR2 RNA and protein analysis by quantitative reverse-transcription PCR and immunohistochemistry

Total RNA (1 μg) extracted with Trizol reagent (Invitrogen) was converted to cDNA using the High Capacity cDNA Reverse Transcription Kit (Applied Biosystems). The PCR reaction was performed by mixing the converted cDNA with the TaqMan Gene Expression Assay for ROR2 (Hs00171695_m1 ROR2) and GAPDH (Hs99999905_m1 GAPDH) and TaqMan Universal PCR Master Mix (Applied Biosystems). qRT-PCR was performed in the Applied Biosystems 7900 HT Fast Real-Time PCR System. Data were normalised using GAPDH as the endogenous control gene. The ROR2 protein was analysed with an anti-ROR2 antibody (from Abcam for immunohistochemistry; from Sigma for WB), following standard procedures.

### ROR2 transfection experiments

The pcDNA3.1-ROR2-Flag construct was created using a pcDNA3.1-ROR2 plasmid (kindly provided by Dr J. Billiard) as a template. The construct was confirmed by DNA sequencing. Control cells were transfected using the empty plasmid (mock). DLD1 cells were transfected with the pcDNA3.1-ROR2-Flag plasmid or the empty plasmid using Lipofectamine 2000 (Invitrogen). Stable transfectants were obtained after 2 weeks selection with 1 mg/ml G418 (Calbiochem). ROR2-Flag expression was confirmed by WB using anti-Flag antibody (1:2000, Sigma).

### Cell viability assay

Cell viability was determined as described [24]. Aliquots (10^4 ^cells) were plated onto 96-well plates. After cell attachment, MTT was added to medium (50 μg; 100 μl/well) and incubated (3 h, 37°C, 5% CO_2_). MTT was removed and MTT-formazan crystals were dissolved in DMSO (100 μl/well). Absorbance at 595 nm was determined with an automated microtitre plate reader. Optical density was directly proportional to cell number up to the maximum density measured. Results are expressed as the mean ± SD of at least five replicates and significance was assessed by standard t-test.

### Colony formation assay

Colony formation was assayed on ROR2-Flag-and mock-transfected DLD1 cells. After 14 days selection, stable G418-resistant colonies were fixed, MTT-stained, and the mean number of colonies in each well was determined.

### Mouse xenograft model

Five-week-old athymic Nude-Foxn1_nu/nu _mice (Charles River) were used for tumour xenograft experiments with ROR2-Flag-and mock-transfected DLD1 cells. Twelve mice were used. Both flanks of each animal were injected subcutaneously with 2 × 10^6 ^cells in 200 μl PBS; ROR2-Flag-transfected cells were injected into the right flank and empty vector cells into the left flank. Mice were weighed, and tumour width (W) and length (L) were measured every 5 days. Tumour volume was estimated according to the formula V = 0.4. L.W^2 ^(L = maximum length; W = maximum width). Mice were killed 30 days post-injection and tumours from both cell types were excised and weighed. Mean volume and tumour mass ± SEM were calculated for each group, and significant differences were assessed by a two-tailed independent-samples t-test.

### Transfection and reporter assays

Subconfluent cultures were transfected in triplicate using JetPEI (PolyPlus Transfection, Illkirch, France) following manufacturer's protocols. pTOPFLASH, and pFOPFLASH reporter plasmids have been described [25]. A *Renilla *luciferase plasmid (pRL-TK) was used in all experiments as an internal control. *Firefly *and *Renilla *luciferase activities were measured separately using the Dual Luciferase reagent kit (Promega, Madison, WI) and a GloMax 96 microplate luminometer (Promega).

### Immunofluorescence

ROR2-Flag-and mock-transfected DLD1 cells were methanol-fixed and stained using mouse anti-β-catenin (Becton Dickinson) and goat anti-α-lamin B (Santa Cruz), followed by Alexa448 donkey anti-goat IgG or Alexa594 donkey anti-mouse IgG (Molecular Probes).

## Competing interests

The authors declare that they have no competing interests.

## Authors' contributions

EL, VC and AFF carried out DNA methylation, expression and transfection experiments and helped to draft the manuscript. CH, AM-P and AJO carried out mouse xenograft experiments. OA and JMG-S carried out reporter assays. LS and AA carried out immunohistochemistry. AM supervised reporter assays and helped to draft the manuscript. CL-O supervised mouse xenograft experiments and helped to draft the manuscript. ME helped to draft the manuscript. MFF conceived the study, participated in its design and wrote the manuscript. All the authors read and approved the final manuscript.
